# Bromide as a Sedative and Hypnotic

**Published:** 1881-09

**Authors:** 


Bromine, as a Sedative and Hypnotic.—Dr. Hammond, of New York, is said to use bromine instead of the alkaline bromides with results equally satisfactory in his practice. This is a therapeutic idea of much value and importance. The beneficial effects of bromine are not marred nor neutralized by such results as acne, ulcers, etc., which the bromide salts sometimes produce. He gives a teaspoonful of a mixture, well diluted, of one drachm of bromine to eight ounces of water, or about one grain of bromine at a dose.

